# Practice patterns and outcomes of direct oral anticoagulant use in myeloproliferative neoplasm patients

**DOI:** 10.1038/s41408-021-00566-5

**Published:** 2021-11-05

**Authors:** Joan How, Charlotte Story, Siyang Ren, Donna Neuberg, Rachel P. Rosovsky, Gabriela S. Hobbs, Jean M. Connors

**Affiliations:** 1 Division of Hematology, Department of Medicine, Brigham and Women’s Hospital, Harvard Medical School, Boston, MA 02115 USA; 2 Department of Medicine, Brigham and Women’s Hospital, Harvard Medical School, Boston, MA 02115 USA; 3 Department of Data Sciences, Dana-Farber Cancer Institute, Boston, MA 02115 USA; 4 Division of Hematology, Department of Medicine, Massachusetts General Hospital, Harvard Medical School, Boston, MA 02114 USA; 5 Department of Medical Oncology, Massachusetts General Hospital, Harvard Medical School, Boston, MA 02114 USA

**Keywords:** Myeloproliferative disease, Medical research

## Abstract

Myeloproliferative neoplasms (MPNs) are characterized by an increased risk of thrombosis and bleeding. Vitamin K antagonists (VKAs) are the historic anticoagulant recommended for use in MPNs. Direct oral anticoagulants (DOACs) are being increasingly used in general and cancer populations. However, DOAC safety and efficacy in MPN patients remains unclear. We characterized real-world practice patterns of DOAC use in MPN patients and evaluated thrombosis and bleeding risk. We conducted a retrospective cohort study of 133 MPN patients prescribed DOACs for venous thromboembolism (VTE), atrial fibrillation, or arterial thromboembolism (ATE). Practice patterns including duration of anticoagulation, dosing, and concomitant use of antiplatelet/cytoreductive agents, were heterogeneous among MPN patients. The 1-year cumulative incidence of thrombosis and bleeding on DOAC was 5.5% (1.5–9.5%) and 12.3% (6.4–18.2%) respectively. In comparison, reported bleeding rates in MPN patients on DOAC and VKAs are 1–3%. On multivariable analysis, prior history of thrombosis, use of dabigatran or edoxaban, and younger age were significantly associated with a higher risk of recurrent thrombosis, while leukocytosis was associated with a higher risk of bleeding on DOAC. The higher-than-expected bleeding rate found in our study indicates the continued need for rigorous evaluation of DOACs in this population.

## Introduction

Myeloproliferative neoplasms (MPNs) are clonal stem cell disorders and include polycythemia vera (PV), essential thrombocythemia (ET), and myelofibrosis (MF). All MPNs have an increased risk of venous thrombosis and cardiovascular events [1]. The *JAK2 V617F* mutation, which is present in nearly all PV patients and approximately 60% of ET and MF patients [2], causes both qualitative and quantitative abnormalities in platelets [3], white blood cells [4], red blood cells [5], and even endothelial cells [6]. Irrespective of *JAK2 V617F* driver mutation status, all MPN patients are characterized by dysregulated Janus kinase (JAK)/signal transducer and activator of transcription (STAT) activation, which further contributes to chronic inflammation and cytokine expression [7]. These factors lead to the hypercoagulable state seen in MPN patients, with the highest incidence of thrombotic events seen in PV (5.5% patients per year), followed by ET (1–3% patients per year) and MF (2% patients per year) [8–10]. Indeed, thrombosis is the leading cause of morbidity and mortality in PV and ET specifically [11].

Management of thrombosis and cardiovascular risk is one of the central therapeutic goals in treating MPN patients. However, current expert consensus guidelines do not include specific recommendations regarding the selection and duration of anticoagulation for the management of vascular events [12]. Vitamin K antagonists (VKAs) are the historic standard anticoagulant recommended for the treatment of thrombosis in MPNs and have been demonstrated to reduce recurrent thrombosis from approximately 9% patients per year to 5% [13–15]. However, VKAs have limitations, including the need for frequent monitoring, numerous dietary restrictions and drug interactions, and a narrow therapeutic window. Direct oral anticoagulants (DOACs) include the direct anti-Xa inhibitors apixaban, rivaroxaban, and edoxaban and the direct thrombin inhibitor dabigatran, and can overcome many of these limitations. Large randomized studies have demonstrated similar efficacy of DOACs compared to warfarin for the treatment of acute VTE or for stroke prophylaxis in atrial fibrillation [16–19], often with decreased bleeding rates compared to VKA. As a result, DOACs are now considered first-line for these indications in the general population [20].

DOAC use has also extended into the general cancer population, with multiple studies demonstrating similar or improved efficacy of DOACs when compared to the previous gold standard, low molecular weight heparin (LMWH), although increased rates of bleeding were seen with some DOACs [21–23]. These trials enrolled few or no MPN patients, making generalizability to this population difficult. In addition, several studies in the general population have demonstrated that extended prophylactic dosing with DOACs for VTE has resulted in similar efficacy rates as therapeutic dosing [24, 25]. However, these strategies may not apply to MPN patients, who have unique risks for thrombosis and bleeding. The duration of anticoagulation for VTE in MPN populations is also unclear, with expert consensus guidelines recommending individualized decision-making [12].

Given these uncertainties and wide practice options in DOAC use, we retrospectively evaluated thrombosis and bleeding risk in a cohort of MPN patients treated with DOACs for a variety of clinical indications. The purpose of this study was to characterize practice patterns and thrombosis/bleeding outcomes of DOAC use in MPNs in a real-world setting. The results of this study are important for generating preliminary data on the safety and efficacy of DOAC use in this population of patients where studies are few and guidelines remain unclear.

## Methods

This study included 133 MPN patients seen across the Massachusetts General Brigham (MGB)/Dana Farber Harvard Cancer Center (DFHCC) from 1986 to 2021. The study protocol was approved by the Institutional Review Board and included a waiver of informed consent for retrospective chart review. Patients were identified using ICD-9 or ICD-10 codes in the electronic medical record. Patients were included if they had a diagnosis of an MPN, including PV, ET, MF, or MPN not otherwise specified (NOS), based on World Health Organization criteria and were prescribed a DOAC (apixaban, rivaroxaban, dabigatran, or edoxaban) [26]. Patients were excluded if a DOAC initiation date could not be found and if DOAC was used for perioperative prophylaxis or prophylaxis during long travel. A total of 133 MPN patients on DOAC were eligible for analysis. We obtained a complete medical history, treatment history, and laboratory data from the electronic medical record for all patients and used summary statistics to describe patient and disease characteristics and practice patterns of DOAC use. Patient characteristics were compared between groups using Wilcoxon rank-sum test for continuous variables and Fisher’s exact test for categorical variables.

Thrombotic and bleeding outcomes in patients were evaluated. A thrombotic event was defined as any venous or arterial thrombosis, as documented by the treating provider. A bleeding event was defined as major bleeding or clinically relevant non-major bleeding (CRNM) by the International Society of Thrombosis and Hemostasis (ISTH) criteria [27]. To evaluate the risk of bleeding or thrombosis on DOAC, only events that occurred on DOAC treatment were counted; in patients that discontinued DOAC during their course, events were not counted if they occurred >3 days after DOAC discontinuation. The time to thrombosis or bleeding was defined as months from DOAC initiation to the occurrence of the event. If patients had their DOAC discontinued or held by their treatment provider, days off treatment were not included. A competing risk model was fitted, with thrombosis or bleeding on DOAC as the event of interest, and death on treatment as the competing event. In competing risk models, we used a cumulative incidence function to describe the risk of an event. A Gray test was used to compare cumulative incidence between patient groups. We then evaluated risk factors for thrombosis or bleeding events using univariate and multivariate Fine–Gray models.

## Results

### Baseline patient characteristics

Baseline characteristics are displayed in Table 1. A total of 133 MPN patients were included in the analysis, of which 75 (56.4%) were on DOAC for VTE, 46 (34.6%) for atrial fibrillation, 7 (5.3%) for stroke, and 5 for other including ATE (3.8%). The median age of patients was 71, and 76 (57%) of patients had a diagnosis of PV; 118 (89%) of all patients carried a JAK2 driver mutation. Apixaban was the most commonly prescribed DOAC (*N* = 83; 62.5%). Nearly half (*N* = 59; 45%) of patients had a prior VTE/ATE at DOAC initiation. Of the 76 patients on DOAC for VTE indication, 20 (26.3%) patients had a prior episode of VTE before being placed on DOAC, and 15 (19.7%) had a prior incident of ATE. In contrast, 3 (6.5%) patients on DOAC for atrial fibrillation had a prior episode of VTE, and 20 (43.4%) patients had a prior episode of ATE. In the entire cohort, 43 (32.3%) had switched to DOAC from a prior anticoagulant with the most common being warfarin (32/43 patients, 74%). Types of VTE events included 39 (43%) deep vein thromboses (DVT), 28 (31%) pulmonary embolism (PE), 19 (21%) splanchnic vein thrombosis (SVT), and 4 (4.4%) other events. Patients with VTE were significantly younger than those with atrial fibrillation (59 vs. 75, *p* < 0.001), more likely to be female (64% vs. 44%, *p* = 0.04), more likely to have prior venous rather than arterial thrombosis (*p* = 0.001), and more likely to have rivaroxaban selected (31.6% vs. 17.4%, *p* = 0.02) with no other significant differences seen in MPN diagnosis, use of anticoagulation prior to DOAC, or JAK2 V617F mutation status.

**Table 1:** Baseline characteristics of MPN patients.

	All	VTE	Afib	Other
*N*	133	75	46	12
Age at DOAC initiation (median, range)	71 (21–95)	66 (21–95)	77 (61–95)	71 (37–92)
*Gender*				
Female	75 (56.4%)	48 (64.0%)	20 (43.5%)	7 (58.3%)
Male	58 (43.6%)	27 (36.0%)	26 (56.5%)	5 (41.7%)
*Race*				
White	117 (88.0%)	65 (86.7%)	43 (93.5%)	9 (75.0%)
Black/African	7 (5.3%)	4 (5.3%)	1 (2.2%)	2 (16.7%)
Asian	4 (3.0%)	3 (4.0%)	1 (2.2%)	0
Hispanic	3 (2.3%)	2 (2.7%)	0 (0)	1 (8.3%)
American Indian/Alaskan Native	1 (0.8%)	0 (0)	1 (2.2%)	0
*MPN subtype*				
PV	76 (57.1%)	42 (56.0%)	25 (54.3%)	9 (75.0%)
ET	35 (26.3%)	19 (25.3%)	13 (28.3%)	3 (25.0%)
MPN NOS	11 (8.3%)	7 (9.3%)	4 (8.7%)	0
Post-ET/PV MF	7 (5.3%)	4 (5.3%)	3 (6.5%)	0
Primary MF	4 (3.0%)	3 (4.0%)	1 (2.2%)	0
*Driver mutation*				
JAK2	118 (88.7%)	68 (90.7%)	39 (84.8%)	11 (91.7%)
CALR	8 (6.0%)	3 (4.0%)	4 (8.7%)	1 (8.3%)
Triple negative	3 (2.3%)	2 (2.7%)	1 (2.2%)	0
MPL	2 (1.5%)	1 (1.3%)	1 (2.2%)	0
Missing	2 (1.5%)	1 (1.3%)	1 (2.2%)	0
*DOAC selected*				
Eliquis/Apixaban	83 (62.4%)	40 (53.3%)	35 (76.1%)	8 (66.7%)
Xarelto/Rivaroxaban	42 (31.6%)	31 (41.3%)	8 (17.4%)	3 (25.0%)
Pradaxa/Dabigatran	7 (5.3%)	3 (4.0%)	3 (6.5%)	1 (8.3%)
Savasya/Edoxaban	1 (0.8%)	1 (1.3%)	0 (0)	0
*Prior history of VTE/ATE*				
No prior VTE/ATE	74 (55.6%)	45 (60%)	24 (52.2%)	5 (41.7%)
Prior ATE	35 (26.3%)	10 (13.3%)	19 (41.3%)	7 (58.3%)
Prior VTE	17 (12.8%)	15 (20%)	2 (4.3%)	1 (8.3%)
Both VTE and ATE	7 (5.3%)	5 (6.7%)	1 (2.2%)	1 (8.3%)
*Thrombophilia*				
FVL	3 (2.3%)	3 (4.0%)	0	0
PGM	3 (2.3%)	3 (4.0%)	0	0
APLS	2 (1.5%)	0	0	2 (16.7%)
PC/PS	1 (0.8%)	1 (1.3%)	0	0
Other	1 (0.8%)	1 (1.3%)	0	0
*Prior anticoagulation*				
Warfarin	31 (23.3%)	18 (24.0%)	9 (19.6%)	4
Fondaparinux	6 (4.5%)	6 (8.0%)	0	0
LMWH	4 (3.0%)	4 (5.3%)	0	0
Other	1 (0.8%)	0	1 (2.2%)	0
BMI at time of DOAC initiation (median, IQR)	27.0 (24.0–30.0)	28.0 (24.0–31.2)	26.0 (24.0–28.0)	25.5 (22.2–34)
Cr at time of DOAC initiation (median, IQR)	1.0 (0.8–1.2)	0.9 (0.7–1.1)	1.0 (0.8–1.2)	1.0 (0.85–1.4)
*CBC at time of DOAC initiation (median, IQR)*				
WBC	10.2 (6.8–15.0)	10.2 (6.3–13.8)	12.0 (7.1–17.2)	7.6 (6.7–36)
HCT	41.0 (36.0–44.8)	40.0 (33.0–44.0)	42.0 (39.4–44.8)	44.9 (36.7–60.0)
PLT	418.5 (264.8–558.0)	380.0 (245.0–556.0)	456.0 (274.0–584.0)	408.5 (278.3–1258.0)
*VTE subtype*				
Proximal DVT	35 (26.3%)			
Distal DVT	4 (3.0%)			
PE	28 (21.1%)			
Splanchnic vein thrombosis	19 (14.3%)			
Cerebral vein thrombosis	1 (0.8%)			
Other venous thrombosis	3 (2.3%)			
*ATE subtype*				
Stroke	8 (6.0%)			
Other arterial thrombosis	4 (3.0%)			

*Afib* atrial fibrillation, *APLS* antiphospholipid antibody syndrome, *ATE* arterial thromboembolism, *BMI* body mass index, *CBC* complete blood count, *Cr* creatinine, *DOAC* direct oral anticoagulant, *DVT* deep vein thrombosis, *ET* essential thrombocythemia, *FVL* factor V Leiden, *HCT* hematocrit, *IQR* interquartile range, *LMWH* low molecular weight heparin, *MF* myelofibrosis, *MPN* myeloproliferative neoplasm, *MPN NOS* myeloproliferative neoplasm not otherwise specified, *PC/PS* protein C/protein S deficiency, *PE* pulmonary embolism, PLT platelets, *PGM* prothrombin gene mutation, PV polycythemia vera, VTE venous thromboembolism, *WBC* white blood cell.

### Practice patterns of DOAC use

The median duration of anticoagulation was 37.0 months in all patients, which was not significantly different (*p* = 0.10) between patients on DOAC for atrial fibrillation (37.0 months) compared to VTE (42.3 months) (Table 2). A total of 20 (12%) of patients completed a finite course of anticoagulation of 6 months median duration. When restricted to VTE patients, 16 (21%) patients completed a finite course of anticoagulation, compared to 3 (6.5%) of atrial fibrillation patients, and 1 patient for stroke. Thirteen patients had a reduction of DOAC dose after a median of 21 months of therapeutic anticoagulation, including 11 (15%) VTE patients and 2 (4.3%) atrial fibrillation patients. At the time of DOAC initiation, half (*N* = 66) of all patients also continued aspirin, and 110 (81%) of patients were also started on or continued cytoreductive therapy. There were 9 patients who interrupted and then resumed DOAC therapy, including 4 patients who completed a recommended course but restarted treatment due to a thrombotic event or physician recommendation, 3 patients who had a bleed and restarted treatment after recovery, 1 patient who had thrombocytopenia and restarted treatment after platelets improved, and 1 patient who had therapy held perioperatively. All treatment discontinuations occurred for 28 days or greater, except for the 1 patient who had DOAC held for 7 days perioperatively.

**Table 2:** Practice patterns of DOAC use.

	All (*N* = 133)	VTE (*N* = 75)	Afib (*N* = 46)	Other (*N* = 12)
Median duration of AC	19.6 months	16.2 months	26.4 months	11.5 months
Finite course	20 (12%)	16 (21%)	3 (58.7%)	1 (50%)
Decreased dose	13 (10%)	11 (21%)	2 (4.3%)	0
Also on ASA	66 (50%)	31 (41%)	29 (63%)	6 (50%)
Also on cytoreduction	110 (82%)	62 (82%)	38 (81%)	10 (83%)

*Afib* atrial fibrillation, *AC* anticoagulation, *ASA* aspirin, *VTE* venous thromboembolism.

### Incidence of thrombosis

**Fig. 1:**
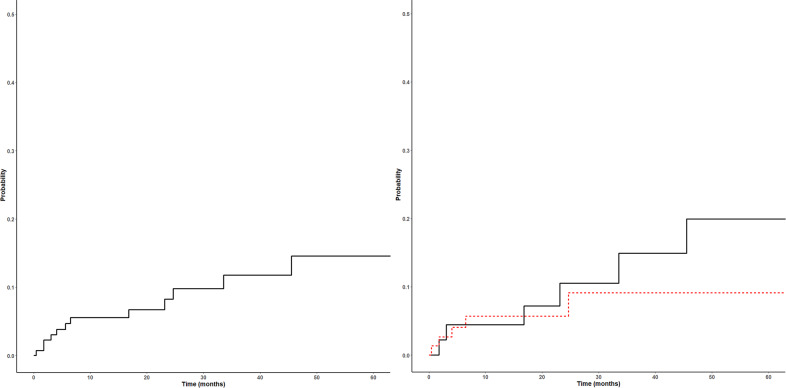
Cumulative incidence of thrombosis on DOAC in all MPN patients (left) and by indication (right). A total of 12 thrombotic events occurred in 133 patients, including 6 thrombotic events in 46 patients on DOAC for atrial fibrillation (black), and 5 thrombotic events in 75 patients on DOAC for VTE (red).

The median follow-up for patients was 37 months. In our cohort, there were 16 thrombotic events, including in 4 patients who were not on DOAC at the time of thrombosis. In these 4 patients, 2 of the recurrent DVTs occurred within 1 year of discontinuing treatment, with the 2 remaining recurrences occurring 1.4 and 3.2 years, respectively. Out of the 12 thrombotic events that occurred on DOAC, 7 were arterial events, 3 were venous events, and 2 were TIPS occlusions. The 1-year cumulative incidence of thrombosis on DOAC was 5.5% (1.5–9.5%) (Fig. 1). Within the MPN patients on DOAC for VTE, there were 5 recurrent thrombotic events, including 2 venous events, 1 arterial event, and 2 TIPS occlusions. In the 6 patients on DOAC for atrial fibrillation, there were 5 arterial events and 1 venous event; 1 patient with a prior CVA had recurrent CVAs despite anticoagulation. There was no significant difference in thrombosis incidence between atrial fibrillation and VTE patients (*p* = 0.47). In the 19 patients treated with DOAC for splanchnic vein thrombosis (SVT), 2 had a TIPS occlusion, with no other thrombotic events. In the 7 recurrent arterial events, 3 patients had been switched from warfarin to DOAC, and in the 3 recurrent venous events, 1 had been switched from warfarin to DOAC; both patients had TIPS occlusions had been switched from either warfarin or LMWH. One patient was on reduced dosing of apixaban for atrial fibrillation at the time of thrombosis, and all others were on therapeutic dosing. Among the 83 patients on apixaban, the most commonly prescribed DOAC, there were 5 recurrent thrombotic events. Eight of the 12 patients were on aspirin concurrently at the time of thrombosis, and 11 of the 12 patients were on cytoreductive therapy.

### Incidence of bleeding

**Fig. 2:**
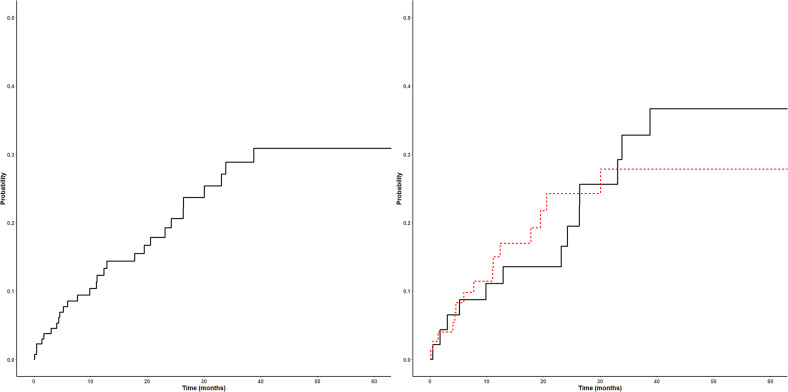
Cumulative incidence of bleeding on DOAC in all MPN patients (left) and by indication (right). A total of 28 bleeding events occurred in 133 patients, including 13 bleeding events in 46 patients on DOAC for atrial fibrillation (black), and 15 bleeding events in 75 patients on DOAC for VTE (red).

There were 29 bleeding events, including 1 patient who was not on DOAC at the time of bleeding. Out of the 28 bleeding events that occurred on DOAC, 6 were major bleeds, including 4 that were resulted in patient death, and 22 were CRNMB. Bleeding events included 11 GI bleeds, 7 hematomas, 3 epistaxis, 3 intracranial, and 3 other bleeds. Within patients treated for DVT, 2 (2.7%) patients had major bleeds, and 13 (17.3%) had CRNMB; within patients treated for atrial fibrillation, 4 (8.7%) had major bleeds, and 9 (20%) had CRNMB. The 1-year cumulative incidence of bleeding on DOAC was 12.3% (6.4–18.2%) (Fig. 2). There was no significant difference in bleeding incidence between atrial fibrillation and VTE patients (*p* = 0.86). In the 19 SVT patients, 5 had bleeding events, including 1 major bleed contributing to patient death, and 4 CRNMB events. Seventeen (60.7%) of these patients were on concurrent DOAC and aspirin at the time of bleed, and 19 (67.9%) were on cytoreductive therapy. Two patients had a second bleed while on DOAC and aspirin. Among the 83 patients on apixaban, the most commonly prescribed DOAC, there were 20 bleeding events. In addition, 8 patients had minor bleeding (4 minor mucosal bleeding including epistaxis, 2 easy bruising, 1 heavy menses, 1 intervention-associated) not meeting CRNMB criteria that resulted in either discontinuation of antiplatelet agents (if prescribed), or a patient-initiated change in therapy (holding therapy or switching DOACs).

### Risk factors for thrombosis and bleeding

We evaluated 16 risk factors for bleeding and thrombotic complications with univariate Fine–Gray modeling, including age, indication for anticoagulation use, prior history of thrombosis, aspirin use, MPN diagnosis, driver mutation, DOAC type and dosing, gender, hematologic parameters at DOAC initiation (white blood cell, hematocrit, and platelet count), body mass index, presence of concomitant thrombophilia, use of cytoreduction, and prior anticoagulation use. Prior history of thrombosis (HR 5.0; 1.4–25.0) and use of dabigatran and edoxaban (HR 5.0; 1.3–19.5) were significantly associated with higher thrombosis risk on univariable analysis, and this remained significant on multivariable analysis (Table 3). Interestingly, age <65 emerged as a significant risk factor for recurrent thrombosis on DOAC in multivariable modeling (HR 3.3; 1.0–10). We found that patients who had white blood cell counts in the top two quartiles of the cohort had a significantly increased risk of bleeding (HR 3.03; *p* = 0.01). There were no other risk factors that predicted bleeding events at the *α* = 0.05 type I level, although there was a trend toward increased bleeding seen with dabigatran or edoxaban (*p* = 0.07).

**Table 3:** Multivariable analysis of significant risk factors associated with recurrent thrombosis on DOAC.

	Hazard ratio (95% CI)	*P*-value
Age at DOAC initiation		
≥65 vs. <65	0.3 (0.1 – 1.0)	0.04
Prior history of thrombosis		
w/o prior clot vs. w/ prior clot	0.1 (0.03 – 0.4)	< 0.001
Type of DOAC		
Xarelto/rivaroxaban vs. eliquis/apixaban	1.3 (0.3 – 5.1)	0.72
Other vs. eliquis/apixaban	6.0 (1.9 – 19.6)	0.003

*DOAC* direct oral anticoagulant.

## Discussion

We evaluated patient characteristics, practice patterns, and thrombosis/bleeding outcomes in a retrospective cohort of 133 MPN patients treated with DOAC. The indications for DOAC use were primarily VTE followed by atrial fibrillation, with 10% of patients treated for arterial events including stroke. Even within our center, there were variations in practice patterns. While most patients remained on indefinite duration anticoagulation, 12% of patients completed a prescribed finite course of anticoagulation. A minority of patients were decreased to prophylactic dosing for secondary VTE prevention after a median of 21 months, similar to the protocol seen in the AMPLIFY-EXTEND and EINSTEIN-CHOICE studies, although this has not yet been studied specifically in MPN patients [24, 25]. There was considerable variability in concomitant use of aspirin, with half of all patients continued on an antiplatelet agent. The use of anti-platelets with anticoagulants in MPN patients is an important aspect of MPN care, given data indicating a higher risk of bleeding with combination therapy [28]. Most patients were on cytoreductive therapy and a DOAC. Cytoreductive therapy is typically recommended in patients who have had a history of thrombosis, but its value in patients with normal cell counts is not defined, a clinical scenario often encountered in young MPN NOS patients who present with SVT, as seen in our cohort.

The cumulative incidence of recurrent thrombosis of 5.5% on DOAC was similar to other studies of DOACs and VKA use in MPNs. Most retrospective studies evaluating anticoagulation in MPNs have included patients on VKAs, with estimated recurrent thrombosis rates of 5.5–9% [13–15, 29]. More recently studies including patients on DOACs have emerged. A meta-analysis of the antithrombotic treatment of VTE in MPN patients pooled 10 studies with a total of 738 MPN patients and found that 22.6% of evaluable patients had a recurrent thrombotic event, including 55 of 313 (18%) on VKA and 5 of 63 (8%) on DOACs [30]. An observational multicenter study of 442 MPN patients on DOACs for either VTE or atrial fibrillation was recently published and found an incidence rate of 4.5% recurrent thromboses per patient year [31]. Similar to this study, we found the majority of recurrent thrombosis events on DOACs were arterial, consistent with greater efficacy of anticoagulation in the venous compared to the arterial system. Our data add to the literature demonstrating similar efficacy of DOAC and warfarin use for the prevention of recurrent thrombosis on anticoagulation.

We found that prior history of thrombosis was a significant risk factor for recurrent thrombosis in multivariate analysis. Lower rates of efficacy with dabigatran and edoxaban were seen compared to apixaban and rivaroxaban, which may be due to specific characteristics of these DOACs. Interestingly, age<65 emerged as a risk factor for recurrent thrombosis on DOAC when included in multivariable modeling. Within ET and PV, prior history of thrombosis and older age are well-established risk factors for thrombosis [32, 33]. However, in our cohort, the higher recurrence rate in younger patients may be driven by the young MPN patients with multiple thrombotic events despite anticoagulation, including MPN NOS patients with abdominal thrombosis and TIPS revisions. Within 19 SVT patients specifically, there were 2 TIPS occlusions without any other thrombotic events. It is difficult to draw conclusions regarding DOAC efficacy in all subpopulations of MPN patients, and DOACs may be less suitable in patients with complex thrombosis history.

However, in contrast to what has been seen previously, we found a much higher risk of bleeding, with a 1-year cumulative incidence rate of 12.3%. In comparison, bleeding rates seen in a recent publication of MPN patients treated on DOACs were 3% in atrial fibrillation and 2.3% in VTE-treated patients [31]. This bleeding risk also appears higher than the bleeding rates reported with VKA use (approximately 1–3%) [15]. Many of the bleeding events were non-trivial and included 6 major bleeding events and 4 that contributed to patient death. The increased incidence of bleeding in our cohort may be related to the higher degree of patient complexity seen at a tertiary referral center. Changes in patient’s renal function, weight, and the addition of interacting medications can also affect bleeding risk during a patient’s course. Declines in glomerular filtration rate (GFR) from DOAC initiation to time of bleeding were noted in at least 6 patients in this cohort, although a limitation of this study was that changes in GFR over time were not collected. Although the decreased renal function is often a consequence of a large bleed, certainly any preceding decline in renal function can exacerbate bleeding risk on DOAC. These variables should therefore be carefully monitored throughout a patient’s course. Bleeding definitions distinguishing CRNMB from minor bleeding may also be subject to interpretation, even when following ISTH criteria, and can vary from study to study. We found separately that an additional 8 patients modified their therapy due to minor bleeding not meeting ISTH criteria. These bleeding events still impact the care of patients as they affect treatment and compliance, and future studies should consider all such events when evaluating anticoagulation safety and efficacy.

These results reinforce the bleeding tendencies seen in the MPN population, and particular care to manage bleeding risk should be taken, especially in complex patients with multiple comorbidities. MPN patients are at increased risk of bleeding for several reasons, although bleeding risk factors are not as well defined compared to risk factors for thrombosis. However, the same abnormalities that can predispose to thrombosis due to abnormalities in hematopoietic cells can also paradoxically lead to increased bleeding. Extreme thrombocytosis (platelets > 1000 × 10^9^/L) has been associated with bleeding due to acquired von Willebrand syndrome (AVWS) as a result of absorption of VWF on platelets, although some postulate that increased cleavage might occur [34]. It is important to note however that AVWS can occur when platelets are below the traditional 1 million thresholds [35]. While we did not find an association between platelet counts and increased bleeding, studies have demonstrated a U-shaped relationship, with increased bleeding at both high and low extremes [36]. The bleeding risk in MPNs is further exacerbated by treatments with both anticoagulants and antiplatelet agents. Although other studies have found that concomitant antiplatelet use is a risk factor for bleeding, we did not find aspirin increased risk of bleeding, which may be a limitation of our sample size and also inconsistent aspirin use during any given patient’s treatment course [28]. Leukocytosis was the only variable found to predict bleeding events in our cohort. Leukocytosis is a well-established risk factor for thrombosis [37], but also has been implicated as a risk for bleeding in a meta-analysis of ET and PV patients [37]. We found a trend toward increased bleeding in patients using edoxaban or dabigatran, similar to findings in Barbui et al. [31]. Optimization of cell counts with cytoreduction and judicious use of concomitant antiplatelet therapy are potential measures to reduce bleeding complications. Monitoring of patients’ renal function and weight, with frequent review of potential interacting medications, is also recommended throughout a patient’s clinical course. Strategies similar to AMPLIFY-EXTEND or EINSTEIN-CHOICE, where extended treatment with lower doses of DOAC is used, have not yet been studied in MPN patients and require further investigation.

In conclusion, we found significantly higher incidence rates of bleeding in MPN patients treated with DOAC compared to previous reports, with similar incidence rates of thrombosis. MPNs are a heterogeneous group of patients with many competing causes of morbidity and mortality, which reflects the different management practices seen within our cohort. The higher-than-expected bleeding rates found in our study indicate the continued need for more rigorous studies of DOACs in this population, with the gold standard being randomized controlled trials comparing DOAC with warfarin.
